# Analysis of CRISPR‐Cas9 screens identifies genetic dependencies in melanoma

**DOI:** 10.1111/pcmr.12919

**Published:** 2020-09-07

**Authors:** Eirini Christodoulou, Mamunur Rashid, Clare Pacini, Alastair Droop, Holly Robertson, Tim van Groningen, Amina F. A. S. Teunisse, Francesco Iorio, Aart G. Jochemsen, David J. Adams, Remco van Doorn

**Affiliations:** ^1^ Department of Dermatology Leiden University Medical Center Leiden The Netherlands; ^2^ Experimental Cancer Genetics Group Wellcome Trust Sanger Institute Cambridge UK; ^3^ Cancer Dependency Map Analytics Wellcome Trust Sanger Institute Cambridge UK; ^4^ Department of Cell and Chemical Biology Leiden University Medical Center Leiden The Netherlands; ^5^ Centre for Computational Biology Human Technopole Milano Italy

**Keywords:** CRISPR‐Cas9 screen, DUSP4, MAPK signaling pathway, melanoma, PPP2R2A

## Abstract

Targeting the MAPK signaling pathway has transformed the treatment of metastatic melanoma. CRISPR‐Cas9 genetic screens provide a genome‐wide approach to uncover novel genetic dependencies that might serve as therapeutic targets. Here, we analyzed recently reported CRISPR‐Cas9 screens comparing data from 28 melanoma cell lines and 313 cell lines of other tumor types in order to identify fitness genes related to melanoma. We found an average of 1,494 fitness genes in each melanoma cell line. We identified 33 genes, inactivation of which specifically reduced the fitness of melanoma. This set of tumor type‐specific genes includes established melanoma fitness genes as well as many genes that have not previously been associated with melanoma growth. Several genes encode proteins that can be targeted using available inhibitors. We verified that genetic inactivation of *DUSP4* and *PPP2R2A* reduces the proliferation of melanoma cells. *DUSP4* encodes an inhibitor of ERK, suggesting that further activation of MAPK signaling activity through its loss is selectively deleterious to melanoma cells. Collectively, these data present a resource of genetic dependencies in melanoma that may be explored as potential therapeutic targets.

**Significance**

Genome‐wide CRISPR‐Cas9 genetic screens in tumor cell lines allow systematic identification of genetic dependencies that may be cancer type‐specific. Here, we analyzed available data from such screens aimed at identifying genes, inactivation of which impairs proliferation of melanoma cells. A subset of the melanoma fitness genes encode proteins that can be inhibited with available small molecule inhibitors. Our data show that for growth melanoma cells not only depend on activators but also on inhibitors of MAPK signaling activity such as *DUSP4*. The melanoma‐specific genetic dependencies identified might be explored as novel specific therapeutic targets.

## 1 INTRODUCTION

Early‐stage cutaneous melanoma can be effectively cured by surgical removal, but once metastasized patient prognosis is poor (Schadendorf et al., 2018). Increased signaling activity of the mitogen‐activated protein kinase (MAPK) pathway is a hallmark of melanoma and can be attributed to mutations in the *BRAF*, *NRAS*, *KIT,* or *NF1* genes. These oncogenic mutations commonly occur in the early stages of melanoma development (Davies et al., 2002; Schadendorf et al., 2018). In patients with *BRAF*‐mutant metastatic melanoma, targeted therapy using BRAF and MEK inhibitors can lead to significant tumor regression. Almost invariably melanoma cells acquire resistance to these targeted treatments, and disease relapse occurs. Consequently, there is a need to identify additional genetic dependencies that might serve as therapeutic targets.

The application of genome‐wide screens in human cancer cell lines has the potential to identify genetic dependencies that may be targeted therapeutically (Barretina et al., 2012; Thompson, Adams, & Ranzani, 2017). Genome editing with CRISPR‐Cas9 technology has improved the identification of genetic dependencies due to its high precision and limited off‐target effects. In CRISPR‐Cas9 dropout screens a population of cells is transduced with a pooled sgRNA library and following culture selective depletion of sgRNAs is measured to identify genes associated with a growth disadvantage or lethal phenotype, designated as fitness genes (Liu & Li, 2019; Shalem et al., 2014). Core fitness genes are involved in essential processes that cells depend on for survival and proliferation. In addition, context‐dependent fitness genes that are specific for cell lineage or genotype are distinguished. Recently, genome‐wide CRISPR‐Cas9 screens have been performed in a range of cancer cell lines, yielding cancer‐specific fitness genes (Bakke et al., 2019; Behan et al., 2019; Dempster et al., 2019; Picco et al., 2019; Tzelepis et al., 2016; T. Wang et al., 2015). Importantly, the majority of cancer‐specific fitness genes were found to be limited to only one or two tumor types (Behan et al., 2019). Specific genetic dependencies in cancer cells may constitute targetable therapeutic vulnerabilities. The objective of this study was to identify novel genetic dependencies that may serve as potential therapeutic targets in melanoma cells through analysis of CRISPR‐Cas9 screen data. Among the 33 fitness genes that we define in melanoma, there are multiple genes that have not previously been associated with melanoma growth, including inhibitors of MAPK signaling activity.

## 2 MATERIAL AND METHODS

### 2.1 CRISPR‐Cas9 screen data

The generation of CRISPR‐Cas9 screen data at Broad Institute, Cambridge Massachusetts, available online at https://depmap.org/ceres/, was described previously (Meyers et al., 2017). Briefly, 341 tumor cell lines including 28 melanoma lines were engineered to express Cas9 and subsequently screened using the human Avana4 library composed of 70,086 sgRNAs, targeting 17,670 protein‐coding genes (4sgRNAs per gene) and 995 non‐targeting control sgRNAs (Meyers et al., 2017). Cancer cell lines were transduced at a multiplicity of infection (MOI) of 0.3 to ensure that each cell expresses only one sgRNA. Genomic DNA was purified from transduced cells cultured under puromycin selection at day 1 and day 21 for next‐generation sequencing. The cell lines included in the screen expressed Cas9.

### 2.2 Pan‐cancer analysis to determine melanoma fitness genes

The CRISPRcleanR package was applied to process the CRISPR‐Cas9‐derived essentiality profiles and to correct for copy‐number amplifications, associated with exacerbated vulnerability scores (Meyers et al., 2017; Iorio et al., 2018). CRISPR‐Cas9 screen data were analyzed using an R implementation of the BAGEL (Bayesian analysis of gene essentiality) algorithm, generating a scaled Bayesian factor (BF) score per gene (Behan et al., 2019; Hart & Moffat, 2016). A 5% false discovery rate (FDR) cutoff was applied. The mutation annotation for each melanoma cell line was derived from the cell line encyclopedia (CCLE) (Ghandi et al., 2019). Gene‐level BFs were computed by calculating the average of the BFs across sgRNAs targeting a gene. This algorithm uses reference sets of predefined essential and non‐essential genes. Each gene was assigned a scaled BF computed by subtracting the BF at the 5% FDR threshold (obtained from classifying reference essential/non‐essential genes using BF rankings) from the original BF. Those genes with a statistically significant depletion at 5% FDR had a scaled BF above zero. Fitness genes were determined by comparing the average dropout of sgRNAs targeting the same gene, to that of reference essential and non‐essential genes (Behan et al., 2019; Hart & Moffat, 2016). Scaled BFs were binarized to 0 (scaled BF<0) and 1 (>0). A Fisher's exact test was performed on a two‐way contingency table of fitness and non‐fitness genes with binarized scaled BF scores from melanoma and the other tumor cell lines with the resulting *p*‐values corrected for multiple testing using the Benjamini–Hochberg procedure (adjusted *p*‐value<.01). Gene expression data for the 28 melanoma cell lines were available for analysis from the CCLE data portal. The data sets are available at https://data.broadinstitute.org/ccle/CCLE_RNAseq_081117.rpkm.gct (Ghandi et al., 2019). Protein interaction networks and pathway enrichment analyses were performed using STRING and Enrichr (Kuleshov et al., 2016; Szklarczyk et al., 2019).

### 2.3 Cell culture, Cas9, and sgRNA lentiviral transduction for validation

Human melanoma cell lines used for functional follow‐up experiments were cultured in DMEM supplemented with 10% fetal bovine serum (FBS), penicillin (100 I.U./ml)/streptomycin (100 μg/ml), and GlutaMAX (Thermo Fisher Scientific). A375, IGR1, and WM983B melanoma cell lines were obtained from ATCC/Rockland, and HEK293T cells were available from lab stocks (LUMC). Cell lines were STR profiled, tested negative for mycoplasma, and cultured in a humidified incubator at 37°C and 5% CO_2_.

Lentivirus for stable Cas9‐expressing cell lines was produced by transfecting pKLV2‐EF1a‐Cas9Bsd‐W (Addgene #68343) into HEK293T cells together with packaging vectors (psPax2 and pMD2.G). For gene inactivation experiments, two sgRNA sequences per gene of interest were cloned into a plasmid DNA vector (U6‐sgRNA‐PGKpuro‐2A‐BFP) from the Sanger CRISPR‐Cas9 genome‐wide arrayed sgRNA library (Table S1) containing the puromycin‐resistance gene for selection (Metzakopian et al., 2017). The sgRNAs used for validations differed from those used in the Broad Avana4 library to provide orthogonal validation. Lentivirus stocks were produced following transfections into HEK293T cells using polyethylenimine (PEI) (Carlotti et al., 2004). Viral titers were determined by antigen capture ELISA measuring HIV p24 (ZeptoMetrix Corp.). Following lentiviral transduction with the Cas9 expression vector to reach a MOI of 3 (100% infection efficiency) in A375, IGR1, and WM983B cells, the Cas9‐editing efficiency was tested (Behan et al., 2019). Capillary sequencing analysis was performed using the human U6 promoter forward primer (GACTATCATATGCTTACCGT) to align the sgRNA sequence to the backbone vector LV04 (Sigma‐Aldrich) and ensure that the sgRNA sequence was correct. For lentiviral transductions with sgRNA expression vectors, 10^5^ cells were seeded in 6‐well plates or 2.5 × 10^5^ cells in 6‐cm dishes. Cells were transduced next day in 2 ml or 5 ml culture medium supplemented with 8 μg/ml polybrene to reach a MOI of 3. Cells were incubated overnight at 37°C and cultured for 4 days in fresh medium supplemented with blasticidin‐S (5 μg/ml) and puromycin (2 μg/ml) for selection and further analysis.

### 2.4 Immunoblot analysis

A375‐Cas9, IGR1‐Cas9, and WM983B‐Cas9 cells were transduced with lentiviral sgRNA expression vectors and cell lysates were prepared for immunoblot analysis as described previously (Christodoulou et al., 2019). Antibodies used for detection included anti‐DUSP4 (1:1,000), anti‐PPP2R2A (1:1,000), anti‐BRAF (1:1,000) (Cell Signaling Technology), and anti‐Vinculin (1:1,000, clone hVIN‐1, Sigma‐Aldrich) as a loading control. Secondary anti‐mouse and anti‐rabbit antibodies were used at 1:10,000 (Jackson ImmunoResearch Europe).

### 2.5 Colony formation assay

After puromycin selection, following transduction with sgRNA expression vectors, the A375‐Cas9, IGR1‐Cas9, and WM983B‐Cas9 cells were trypsinized, counted, and seeded in triplicate in 12‐well plates at a density of 500 cells/ml (A375), 2,000 cells/ml (IGR1), and 3,000 cells/ml (WM983B) for colony formation assays. Cells were fixed for 5–10 min in 4% paraformaldehyde (PFA) and stained for 30 min with crystal violet (0.05%) when control wells transduced with negative control sgRNAs targeting *SSX3* reached 80% confluency. Scanned images of the wells were obtained before solubilization of retained dye with 100% methanol to measure absorbance at OD540.

### 2.6 Cell confluency assay

IGR1‐Cas9 and A375‐Cas9 cells were seeded in 96‐well plates to monitor cell confluency using the IncuCyte live‐cell analysis system (Essen BioScience). For this assay cells were seeded at a density of 50 cells/well (A375) and 200 cells/well (IGR1) in 6 replicates and scanned images of each well were taken every 12 hr. Data were analyzed using IncuCyte software and confluency was calculated over 6 days (A375) and 8 days (IGR1) and normalized to the confluency on day 1 to correct for seeding variation. Colony formation and cell confluency assays were performed in biological duplicates, and combined data were analyzed using GraphPad Prism version 8. A 2‐way ANOVA and Bonferroni's multiple comparisons test were performed to detect statistically significant differences in cell confluency (*p*‐value<.01).

## 3 RESULTS

### 3.1 CRISPR‐Cas9 screen analysis for fitness genes in melanoma

To identify genes that are specifically required for the fitness of melanoma cells, data available from CRISPR‐Cas9 screens performed at the Broad Institute (Cambridge, Massachusetts) were extensively processed and analyzed. In these genome‐wide CRISPR‐Cas9 dropout screens, the depletion of sgRNAs from a genome‐wide library was measured in populations of Cas9‐expressing tumor cells following 21 days of culture after transduction (Meyers et al., 2017). Fitness genes were determined by comparing the average dropout of sgRNAs targeting the same gene to the profile of reference essential and non‐essential genes using a supervised approach called BAGEL (Behan et al., 2019; Hart & Moffat, 2016). Of all targeted genes, 4,423 (25%) negatively affected the fitness of one or more melanoma cell lines. We found an average of 1,494 fitness genes in each of the 28 melanoma cell lines. There were 1,396 genes that reduced the fitness in half or more of the melanoma cell lines, a number similar to what was reported for other cancer types (Behan et al., 2019). Our analysis yielded 193 genes that reduced fitness in all melanoma cell lines, 175 of which (91%) had been found to be core fitness genes in the haploid cell line HAP1 and are not specific to melanoma (Table S2) (Blomen et al., 2015).

**Figure 1:**
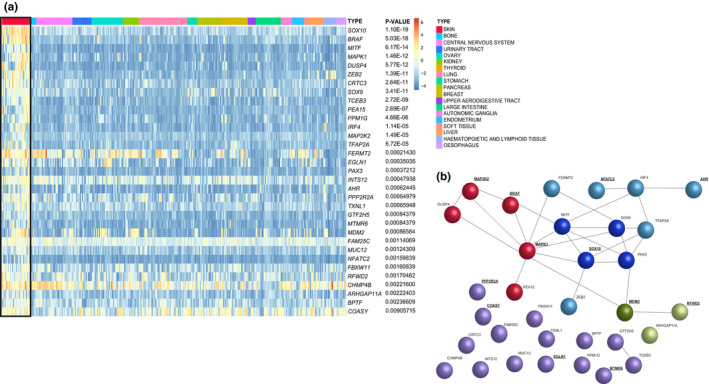
Identification of genetic dependencies in cutaneous melanoma cells. (a) Heatmap representation of genes significantly associated with cellular fitness in melanoma cell lines indicated in a box. The scale bar represents scaled Bayesian factors (BFs) within each screen, calculated by subtracting the BF at the 5% FDR threshold, obtained when classifying prior known essential/non‐essential genes based on their BF’s rank (Behan et al., 2019). Red color indicates genes that are likely to be fitness genes and, therefore, have a positive scaled BF. Blue color indicates genes less likely to be important for cell fitness with a negative scaled BF. Tumor types are shown and clustered. Genes were ranked according to the noted Fisher exact test‐adjusted *p*‐values. (b) String protein interaction network for 33 significant fitness genes in melanoma cell lines. Color coding: Red—proteins involved in the MAPK signaling pathway; blue—melanocyte‐lineage‐specific proteins; green—p53‐regulatory pathway components; purple—unknown interaction network, miscellaneous function; dark—core protein components; light—regulatory components. For proteins indicated in underlined‐bold text pharmacological inhibitors are currently available

**Figure 2:**
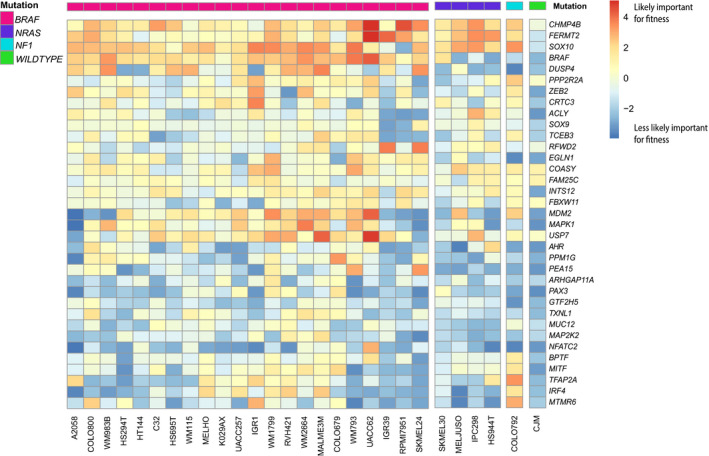
Significant fitness genes in 28 melanoma cell lines. Heatmap representation of fitness genes specifically important in cutaneous melanoma cell lines and indication of established driver mutations (*BRAF, NRAS, NF1*) for each cell line. Supervised clustering was performed on columns to distinguish clusters of *BRAF, NRAS, and NF1‐*mutant and wild‐type cell lines. The genes are ranked according to fitness effect that is denoted by the scaled BF score within the set of 28 melanoma cell lines. Genes that are high on the heatmap had a more positive scaled BF score (red) indicating that are more likely to be important for fitness, whereas genes that are low on the heatmap (blue) had a more negative scaled BF score indicating that are less likely to be important for fitness

To identify tumor type‐specific genetic dependencies for melanoma, we performed comparative analysis of the scaled BFs representing fitness effects for each gene in the 28 melanoma cell lines and in the 313 cell lines from 18 other tumor types analyzed in parallel in the CRISPR‐Cas9 screens (Behan et al., 2019; Meyers et al., 2017). CRISPR‐mediated inactivation of 33 genes was significantly associated with reduced fitness in melanoma when compared to tumor cell lines from other lineages after multiple testing correction (Figure 1a). The average of median RPKM values of 31/33 abovementioned genes was 14,606 (Q1 = 9,997 and Q3 = 20,103) (Figure S1). All *BRAF*‐mutant melanoma cell lines showed reduced survival upon targeting of *BRAF*. The screen identified melanoma fitness genes having an effect size similar to *BRAF*, including *CHMP4B, FERMT2* and *DUSP4* supporting their potential as therapeutic targets (Figure 2). Of the 28 melanoma cell lines, 22 harbored *BRAF* V600 mutations, 4 harbored oncogenic *NRAS* mutations, one was *NF1‐*mutant (in the absence of *BRAF* or *NRAS* mutations), and the remainingline was triple‐wild‐type. There were no *KIT* mutations in any of the tested melanoma cell lines (Figure 2, Supplemental Table S3). This set of 33 fitness genes related to melanoma included melanocyte‐specific transcription factors such as *MITF* and *SOX10*, established as fitness genes for cells of the melanocytic lineage (Figure 1b, Table S4) (Hemesath et al., 1994; Shakhova et al., 2012). A second cluster of fitness genes related to melanoma consisted of components of the MAPK signaling pathway, such as *BRAF* and *MAPK1* (Table S5). The finding of these established fitness genes in this gene set confirms the sensitivity of the CRISPR‐Cas9 screen analysis. A third group of genes, to which *MDM2* belongs, is involved in regulating p53 activity (Table S4). For several genes, a role in melanoma progression has been reported; *FERMT2* for instance has been found to impact melanoma metastasis (Karras et al., 2019). Moreover, activation of *AHR* was reported to promote resistance to BRAF inhibitors in melanoma (Corre et al., 2018). In addition, a set of fitness genes related to melanoma with undefined roles in melanocyte biology or melanoma pathogenesis was identified, such as *MTMR6* and *CRTC3*. Pharmacological compounds are available that may halt melanoma growth for the products of 12 of the identified fitness genes related to melanoma, including *AHR* and *MDM2* (Table S4).

### 3.2 Negative regulators of MAPK signaling are fitness genes in melanoma

Remarkably, the identified MAPK signaling genes encoded not only activating but also inhibitory components such as *DUSP4* and *PEA15*. DUSP4 dephosphorylates ERK1/2, in addition to p38 and JNK (Table S5) (Hutchinson et al., 2015), while PEA15 is a negative regulator of MAPK signaling that acts by sequestering ERK1/2 in the cytoplasm (Shin, Lee, Yang, Jeon, & Song, 2015). The protein phosphatase PPP2R2A has pleiotropic functions, including regulation of ERK1/2 and AKT phosphorylation and is involved in double‐strand DNA repair (Table S5) (Kalev et al., 2012; Martina & Puertollano, 2018; Omerovic, Clague, & Prior, 2010; Shouse, de Necochea‐Campion, Mirshahidi, Liu, & Chen, 2016). As melanoma is characterized by activation of MAPK signaling due to mutations in *BRAF, NRAS, NF1* and *KIT* genes, this suggests that further hyperactivation of MAPK signaling through loss of inhibitors of this signaling pathway may be deleterious to melanoma cells. Based on their melanoma‐specificity (*p*‐value), effect size (scaled BF score), and gene function, we proceeded with functional in vitro studies of two genes encoding for proteins with a reported inhibitory effect on MAPK signaling activity, *DUSP4* and *PPP2R2A*. *DUSP4* was a significant fitness gene in 16 melanoma cell lines including one *NRAS*‐mutant, and *PPP2R2A* significantly affected fitness of 16 melanoma cell lines, including one *NRAS*‐mutant and one *NF1*‐mutant (Supplemental Figure 1, Figure 2). *DUSP4* and *PPP2R2A* were homogenously expressed among the 28 melanoma cell lines and transcript levels did not provide an explanation for the difference in sensitivity to inactivation—this might imply post‐translational factor influence on the function of these genes.

We used CRISPR‐mediated inactivation of the non‐fitness gene *SSX3* as a negative control, and as the cell lines used for validation were *BRAF*‐mutant, inactivation of *BRAF* was used as a positive control. First, we analyzed the effects of depletion of the candidate genes in the *BRAF*‐mutant melanoma cell line WM983B that was included in the CRISPR‐Cas9 screens performed by the Broad institute. Proteins encoded by the target genes *BRAF*, *DUSP4* and *PPP2R2A* were significantly depleted by CRISPR‐Cas9‐mediated inactivation using two independent sgRNAs, as was confirmed by immunoblot analysis 4 days after transduction (Figure S2a). The sgRNAs against *BRAF* also led to a decrease in DUSP4 protein levels, in accordance with regulation of DUSP4 by MAPK signaling as part of a negative feedback loop (Hutchinson et al., 2015). Upon CRISPR‐mediated inactivation of *DUSP4* and *PPP2R2A,* a significant effect on WM983B cell viability was observed as measured using the crystal violet assay (Figure S2b). Loss of proliferation caused by *DUSP4*‐mediated depletion was similar to *BRAF*‐mediated depletion in WM983B cells (Figure S2c). The effects of inactivation of *PPP2R2A* using both sgRNAs were slightly less pronounced in this cell line. Combined, these data suggest that genetic depletion of *DUSP4* and *PPP2R2A* has a significant effect on the proliferation of WM983B cells.

**Figure 3:**
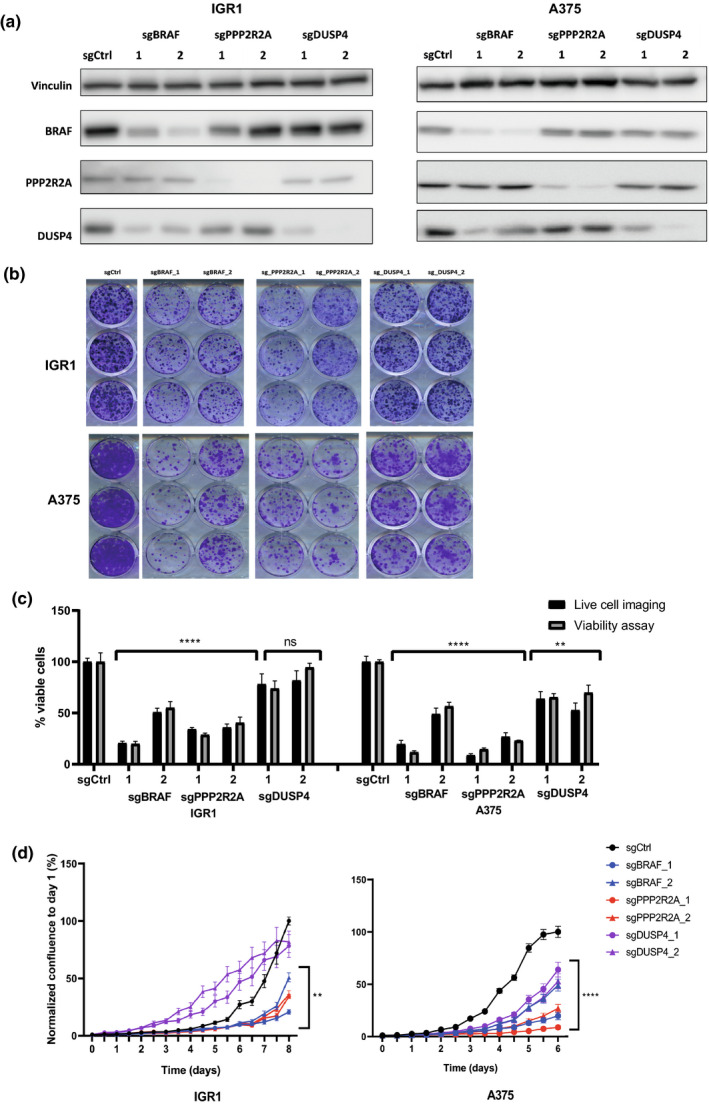
Validation of CRISPR‐mediated inactivation of *DUSP4* and *PPP2R2A* on proliferation of IGR1 and A375 cutaneous melanoma cell lines. (a) Immunoblot analysis of BRAF, PPP2R2A, and DUSP4 protein expression 4 days after transduction with sgRNA expression vectors in IGR1 and A375 cells. Expression of vinculin was investigated as a loading control. (b) Images of crystal violet assays of a control depletion (*SSX3*), *BRAF,* and two independent sgRNAs for *PPP2R2A* and *DUSP4* in IGR1 and A375 cells. (c) Comparison of two independent techniques, live‐cell imaging in 96‐well plates and cell viability assay in 12‐well plates for different knockout IGR1 and A375 lines. A 2‐way ANOVA and Bonferroni's multiple comparisons test was performed between the two techniques (*p<.01). Error bars represent *SE* of the mean. Two independent experiments were performed. (d) Graphical representation of cell proliferation up to 8 days after seeding of IGR1 cells and 6 days after seeding of A375 cells. sgCtrl represents depletion of *SSX3*, *BRAF* depletion was used as a positive control, and 2 independent sgRNAs were used for *PPP2R2A* and *DUSP4*. The normalized confluency (%) of IGR1 and A375 cells was corrected based on day 1 measurements. A 2‐way ANOVA and Bonferroni's multiple comparisons test was performed between the control and all other lines (*p<.01)

Next, we examined the effects of *DUSP4* and *PPP2R2A* depletion in two additional melanoma cell lines, both harboring *BRAF* mutations. Whereas the IGR1 melanoma cell line was also included in the CRISPR‐Cas9 screens, the A375 melanoma cell line was not. Immunoblot analysis confirmed depletion of *PPP2R2A* and *DUSP4* upon sgRNA transduction (Figure 3a). We could verify that *PPP2R2A* inactivation affects the viability of IGR1 cells 8 days after seeding according to the colony formation assay (Figure 3b), in accordance with results from CRISPR‐Cas9 screen analysis. When monitoring cell confluency over time, we observed decreased proliferation upon *PPP2R2A* inactivation in IGR1 cells, in line with the colony formation data (Figure 3c,d). As for the independent A375 cell line, significant loss of viability was confirmed upon inactivation of *DUSP4* and *PPP2R2A* through the colony formation assay (Figure 3b,c) and by normalized cell confluency over time (Figure 3d). Loss of proliferation upon CRISPR‐Cas9‐mediated inactivation of *PPP2R2A* was stronger when compared to *DUSP4* depletion using both sgRNAs in A375 cells. One sgRNA targeting *BRAF* was more effective than the other in reducing cell viability and proliferation in all tested *BRAF*‐mutant cell lines (Figure 3). Further studies are needed to unravel the nature and mechanism of the effects underlying these genetic dependencies. Decreased protein levels of DUSP4 and PPP2RA were detected in all melanoma cell lines by immunoblot analysis up to 10 days after transduction (Figure S3) confirming the effects of *DUSP4* and *PPP2RA* on cellular fitness in melanoma cell lines. This suggests dependency of genes encoding inhibitors of oncogenic kinome signaling.

## 4 DISCUSSION

Here, we present the results of analysis of genome‐wide CRISPR‐Cas9 screens aimed at identifying genes that melanoma cells depend on for their fitness. The comparative analysis of the effects on fitness of gene inactivation in melanoma cells and other tumor cell lines uncovered a set of 33 fitness genes related to melanoma. We verified the fitness effects of *DUSP4* and *PPP2R2A* in multiple melanoma cell lines. Genetic inactivation of several identified fitness genes had an effect on melanoma cell proliferation similar in size to *BRAF* inactivation. Many of the identified fitness genes such as *NFATC2* and *EGLN1* have an as yet undetermined role in melanoma biology. For 12 proteins encoded by genes identified as essential in the present study, pharmacological compounds are available, implying that certain existing drugs might be efficacious in the treatment of melanoma.

Strengths of this study are the sensitivity and robustness of the CRISPR‐Cas9 screening methodology by targeting all protein‐coding genes using 4 sgRNAs per gene and data analysis through normalization of copy number‐associated effects, as well as comparative analysis of genetic dependencies in 28 melanoma cell lines with those of 313 other tumor cell lines. Evaluation of CRISPR‐Cas9 screens in human melanocytes would have allowed further delineation of genes that are essential in melanoma from lineage‐specific fitness genes, but such data are not yet available. As our identified hits include the melanocytic lineage transcription factors *MITF* and *SOX10*, it is probable that some of the other genes affect melanocyte fitness as well. Eleven of the identified 33 genes have been previously identified as fitness genes in haploid, CML‐derived HAP1 cells (*BRAF, MAPK1, ZEB2, TCEB3, FERMT2, PPP2R2A, BPTF CHMP4B, INTS12, FBXW11,* and *COASY*) (Blomen et al., 2015). Inactivation of these genes is significantly more detrimental to melanoma cells than to other tumor cell types, but they are likely to be involved in essential cellular processes. The composition of the fitness gene set related to melanoma may be determined in large part by dependencies associated with mutant *BRAF*. In a previous study, gene dependency associations with *BRAF* mutation have been identified in 16 cancer types (Dempster et al., 2019). Mutant *BRAF* was present in nine of 146 of cancer cell lines, and this oncogenic mutation was found to be associated with dependency on 50 genes in these cancer types.

We demonstrate genetic dependency on multiple MAPK signaling components in melanoma. Interestingly these include not only activators but also inhibitors of MAPK signaling, such as DUSP4 and PEA15. DUSP4 regulates phosphorylation of ERK, but also of p38, JNK and other proteins (Mazumdar et al., 2016). *DUSP4* depletion was recently reported to diminish the negative effects on melanoma cell viability induced by MEK inhibitors through increasing MAPK activity (Gupta et al., 2020). Our results support the notion that further activation of MAPK signaling through loss of inhibitors such as DUSP4 in melanoma cells that already harbor activating *BRAF* or *NRAS* mutations is detrimental, a hypothesis that will need to be explored with further experiments. Accordingly it has been reported that ERK1 and ERK2 overexpression results in cell death in *BRAF* and *NRAS*‐mutant melanoma cells (Leung et al., 2019). Most melanoma cell lines included in the CRISPR‐Cas9 screens carried the *BRAF* mutation, but screen data suggested that some *NRAS‐*mutant melanoma cells might also be sensitive to inactivation of *DUSP4* and *PPP2R2A*. Targeting these proteins may provide an alternative approach to treatment of metastatic melanoma, particularly after relapse from immunotherapy or targeted therapy. Acquisition of resistance to BRAF inhibitors commonly involves reactivation of MAPK signaling. Discontinuation of BRAF inhibitor treatment in resistant melanoma cells results in hyperactivated MAPK signaling, which may be detrimental to these cells (Kong et al., 2017; Sun et al., 2014). We hypothesize that once melanoma cells have acquired resistance to treatment with BRAF and MEK inhibitors by upregulation of MAPK activity they will become more sensitive to *DUSP4* inhibition. Targeting negative regulators of MAPK signaling inhibitors such as DUSP4 and PEA15 further activates MAPK signaling and may therefore be particularly effective in eliminating melanoma cells that have acquired resistance to BRAF and MEK inhibition. Alternate treatment with BRAF inhibitors and inhibitors targeting negative regulators of MAPK signaling could constitute an effective treatment strategy. The lack of specific pharmacological inhibitors for DUSP4 and PEA15 currently limits the possibility to determine those effects.

Genetic inactivation of *DUSP4* significantly reduced proliferation in a melanoma cell line that was included in the CRISPR‐Cas9 screens performed by the Broad institute (WM983B) as well as an independent melanoma cell line (A375). This suggests that DUSP4 could be an effective target across a larger panel of melanoma cell lines.Consistent with findings from the CRISPR‐Cas9 screens, inactivation of *DUSP4* did not induce a growth disadvantage in the IGR1 cell line, showing heterogeneity between different melanoma cell lines with respect to fitness effects. In addition, not only *BRAF*‐mutant but some *NRAS*‐mutant melanoma cell lines were sensitive to inhibition of *DUSP4* and *PPP2R2A. PPP2R2A* was confirmed to be a fitness gene in the two cell lines included in the initial CRISPR‐Cas9 screens, IGR1 and WM983B as well as in the independent A375 cell line. PPP2R2A is a PP2A regulatory subunit that has been reported to inhibit MAPK signaling by dephosphorylating ERK (Koutsioumpa et al., 2018), but also to promote MAPK signaling by regulating RAF and KSR (Hein et al., 2016; Ory, Zhou, Conrads, Veenstra, & Morrison, 2003). The PP2A complex has broader cellular functions, including regulation of oxidative stress signaling and DNA repair response (Martina & Puertollano, 2018; Omerovic et al., 2010; Q. Wang et al., 2016; Yan et al., 2012). We have not investigated whether the fitness effects of DUSP4 and PPP2R2A are strictly dependent on their function as inhibitors of oncogenic kinome signaling.

The generation of effective treatment strategies in melanoma remains a challenge. Here, we present an analysis of CRISPR‐Cas9 screen data aimed at melanoma cell lines, identifying 33 genes that specifically affect the fitness in this tumor type. In vitro experiments in human melanoma cell lines confirmed that inactivation of *DUSP4* and *PPP2R2A* results in decreased cell proliferation. Collectively, these data present a resource of genetic dependencies in melanoma that may be explored as potential therapeutic targets.

## CONFLICT OF INTEREST

The authors declare that there is no conflict of interest to disclose.

## Supporting information

Fig S1

Fig S2

Fig S3

Table S1

Table S2

Table S3

Table S4

Table S5

Supplementary Material
