# Supplementary figures and images for: Analysis of CRISPR‐Cas9 screens identifies genetic dependencies in melanoma
Source: Pigment Cell Melanoma Res. 2020 Sep 7;34(1):122–31. doi: 10.1111/pcmr.12919 (PMC7818247; doi:10.1111/pcmr.12919)

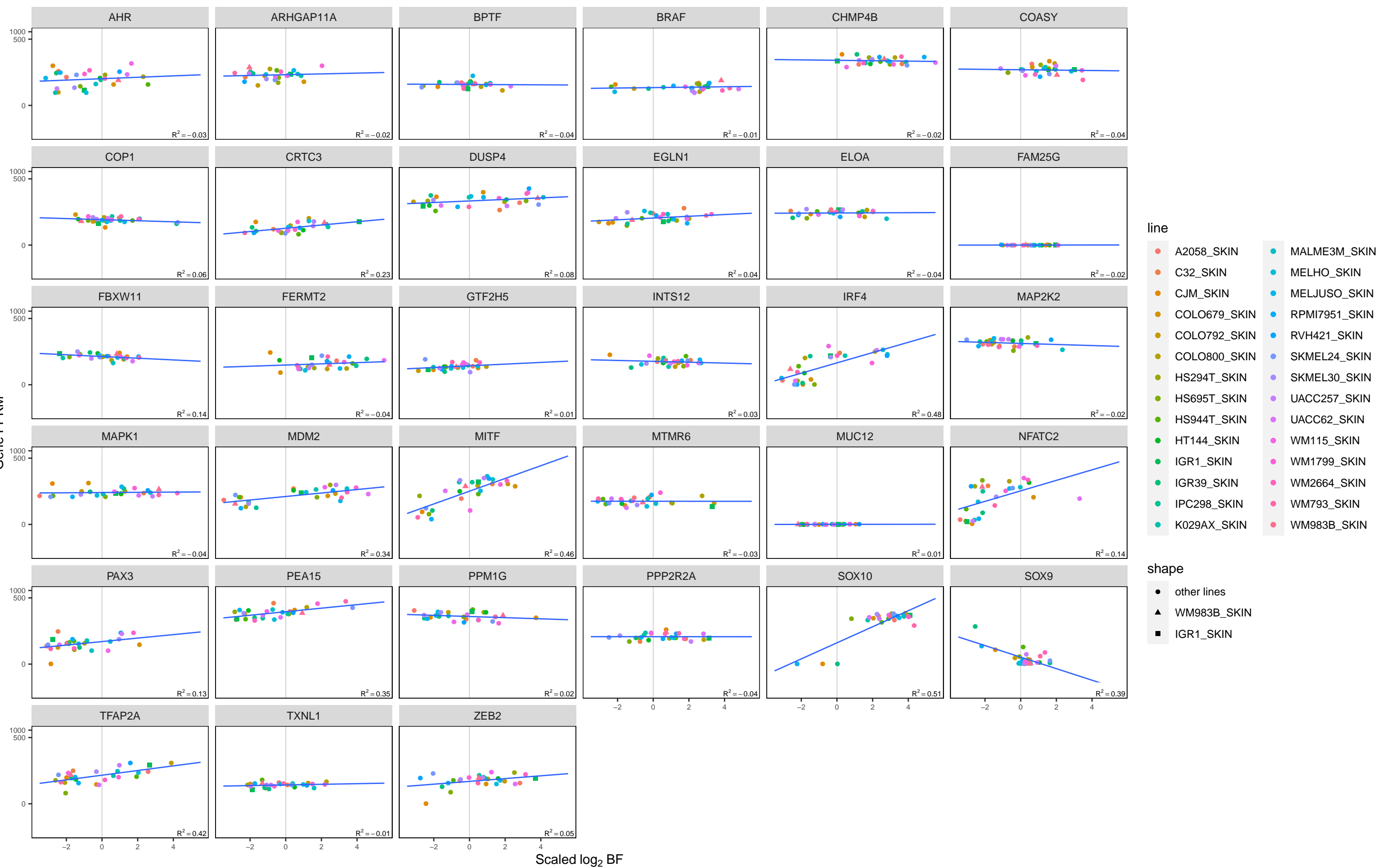

Supplement: Supplementary file 1 — Fig S1 [file PCMR-34-122-s001.pdf]

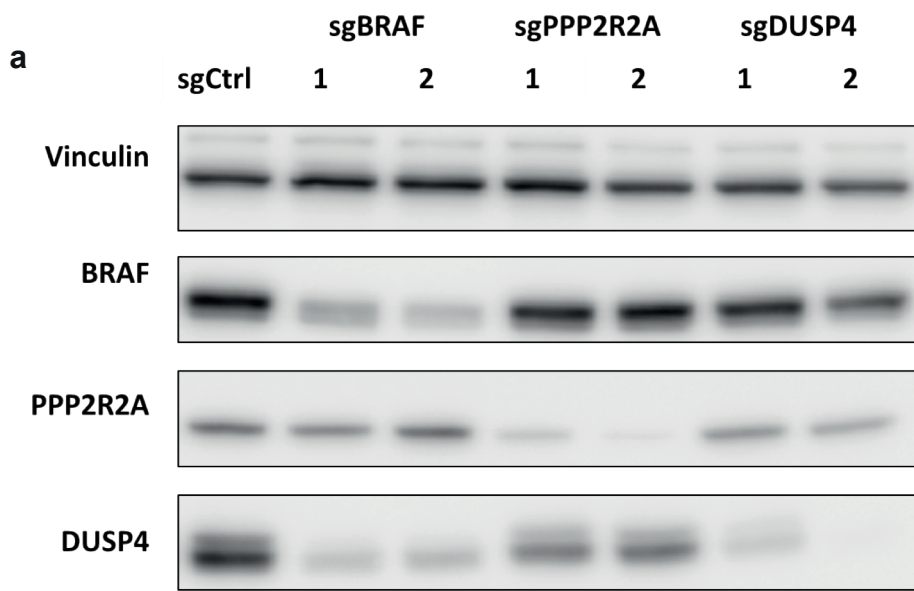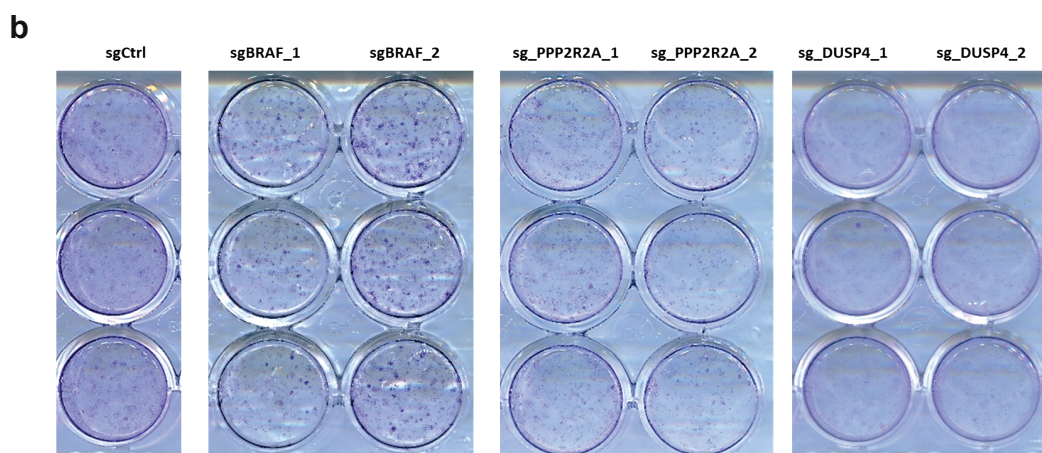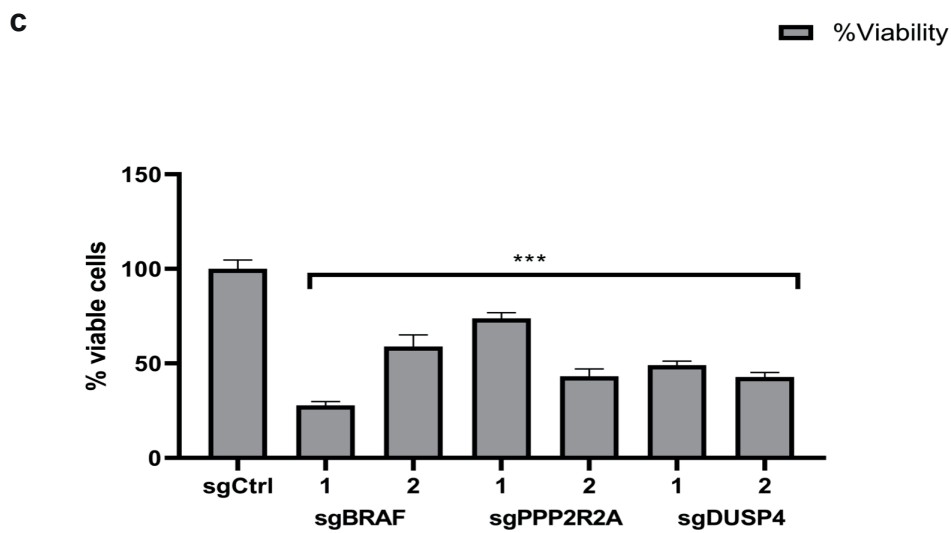

Supplement: Supplementary file 2 — Fig S2 [file PCMR-34-122-s002.pdf]

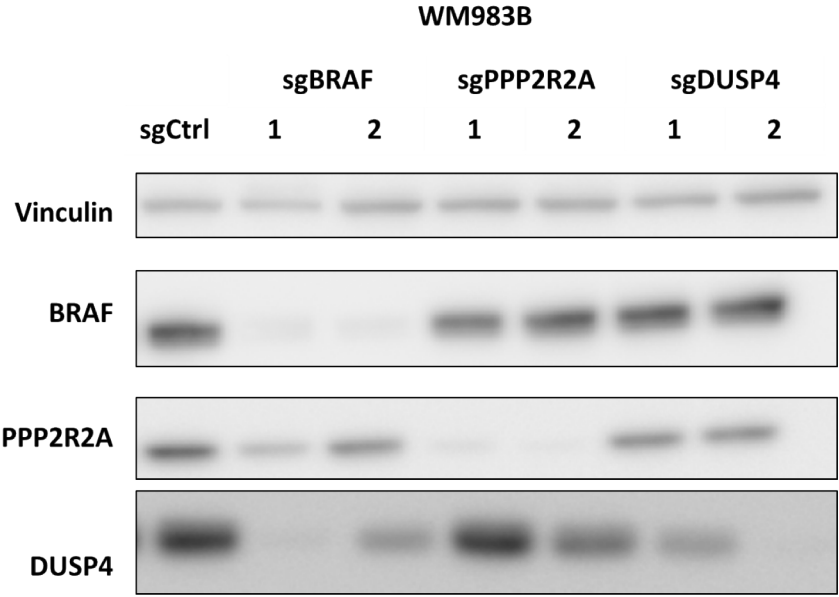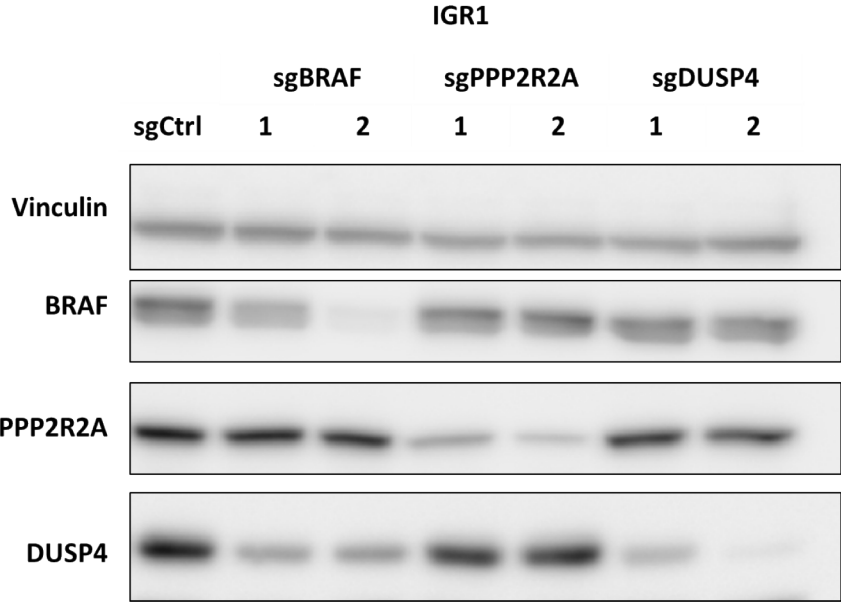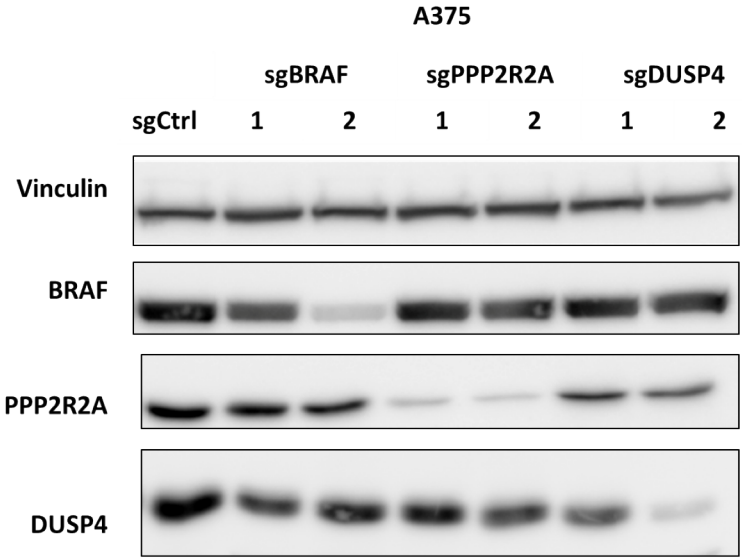

Supplement: Supplementary file 3 — Fig S3 [file PCMR-34-122-s003.pdf]
