# Supplementary material for: Analysis of CRISPR‐Cas9 screens identifies genetic dependencies in melanoma
Source: Pigment Cell Melanoma Res. 2020 Sep 7;34(1):122–31. doi: 10.1111/pcmr.12919 (PMC7818247; doi:10.1111/pcmr.12919)
Supplement: Supplementary file 9 — Supplementary Material [file PCMR-34-122-s009.docx]

## **SUPPORTING INFORMATION**

## **Figure legends**

**Figure 1**

**Identification of genetic dependencies in cutaneous melanoma cells** **a)** Heatmap representation of genes significantly under-represented in melanoma cell lines indicated in a box. The scale bar represents scaled Bayesian factors (BFs) within each screen, calculated by subtracting the BF value corresponding to the threshold guaranteeing a 5% FDR obtained when classifying prior known essential/non-essential genes based on their BF’s rank (Behan et al., 2019). Red color indicates genes that are likely to be fitness genes and, therefore, have a positive scaled BF. Blue color indicates genes less likely to be important for cell fitness with a negative scaled BF. Tumor types are shown and clustered. Genes were ranked according to the noted Fisher exact test adjusted p-values. **b)** String protein interaction network for 33 significant fitness genes in melanoma cell lines. Color coding: Red- proteins involved in the MAPK-signaling pathway; blue- melanocyte-lineage specific proteins; green- p53-regulatory pathway components; purple- unknown interaction network, miscellaneous function; dark- core protein components; light- regulatory components. For proteins indicated in bold pharmacological inhibitors are currently available.

**Figure 2**

**Significant fitness genes in 28 melanoma cell lines**. Heatmap representation of fitness genes specifically important in cutaneous melanoma cell lines and indication of established driver mutations (*BRAF, NRAS, NF1*) for each cell line. Supervised clustering was performed on columns to distinguish clusters of *BRAF, NRAS, NF1* mutant and wildtype cell lines. The genes are ranked according to fitness effect that is denoted by the scaled BF score within the set of 28 melanoma cell lines. Genes that are high on the heatmap had a more positive scaled BF score (red) indicating that are more likely to be important for fitness whereas, genes that are low on the heatmap (blue) had a more negative scaled BF score indicating that are less likely to be important for fitness.

**Figure 3**

**Validation of *PPP2R2A* and *DUSP4* knockout on proliferation of IGR1 and A375 cutaneous melanoma cell lines**. **a)** Immunoblot analysis of BRAF, PPP2R2A and DUSP4 protein expression 4 days post-transduction with sgRNA expressing lentiviruses in IGR1 and A375 cells. Expression of vinculin was investigated as a loading control. **b)** Crystal violet images of a control depletion (*SSX3*), *BRAF* and two independent sgRNAs for *PPP2R2A* and *DUSP4* in IGR1 and A375 cells*.* **c)** Comparison of two independent techniques, live cell imaging in 96-well plates and cell viability assay in 12-well plates for different knockout IGR1 and A375 lines. A 2-way ANOVA and Bonferroni’s multiple comparisons test was performed between the two techniques (*p<0.01). Error bars represent SE. **d)** Graphical representation of cell proliferation up to 8-days post seeding of IGR1 cells and 6-days post seeding of A375 cells. sgCtrl represents depletion of *SSX3*, *BRAF* depletion was used as a positive control and 2 independent sgRNAs were used for *PPP2R2A* and *DUSP4*. The normalized confluency (%) of IGR1 and A375 cells was corrected based on day 1 measurements. A 2-way ANOVA and Bonferroni’s multiple comparisons test was performed between the control and all other lines (*p<0.01).

**Supplemental material**

**Supplemental Figure 1**

**Expression of 33 significant fitness genes in melanoma cell lines.** The scaled Bayesian factor score is plotted against the FPKM (expression) score for each gene using data from CCLE. The FPKM is shown on a “pseudo-log” axis. The blue line is a simple linear correlation model with the adjusted R-squared value shown. The vertical grey line shows the fitness switch; the left-hand genes are non-fitness, but to the right are most probably fitness genes. All “SKIN” cell lines are melanoma cell lines.

**Supplemental Figure 2**

**Depletion of *PPP2R2A* and *DUSP4* has a significant effect on WM983B proliferation**. **a)** Immunoblot analysis of BRAF, PPP2R2A and DUSP4 protein expression 4 days post-transduction with sgRNA expressing lentiviruses. Expression of vinculin was investigated as a loading control. **b)** Crystal violet images of a control depletion (*SSX3*), *BRAF* and two independent sgRNAs for *PPP2R2A* and *DUSP4.* **c)** Graphical representation of cell viability in WM983B cells 10-days post seeding. sgCtrl represents depletion of *SSX3*, *BRAF* depletion was used as a positive control and 2 independent sgRNAs were used for *PPP2R2A* and *DUSP4*. Experiments were performed in two biological replicates and in Figures 3-5 we show results of a representative experiment. A 2-way ANOVA and Bonferroni’s multiple comparisons test was performed between the control and all other lines (*p<0.01). Error bars represent SE.

**Supplemental Figure 3**

**Immunoblot analysis in tested melanoma cell lines.** Immunoblot analysis of BRAF, PPP2R2A and DUSP4 protein inhibition 10 days post-transduction in all tested melanoma cell lines (A375, IGR1, WM983B). Expression of vinculin was investigated as a loading control.

**Supplemental Table 1**

**Sequences of gRNAs used for single gene validation analysis.**

**Supplemental Table 2**

**Somatic mutations in 28 melanoma cell lines** Data obtained from cell line encyclopedia (BROAD institute) (Ghandi et al., 2019).

**Supplemental Table 3**

**List of melanoma-related fitness genes in haploid cells** (Blomen et al., 2015)

**Supplemental Table 4**

**Description of 33 significant fitness genes in melanoma**

**Supplemental Table 5**

**Pathway enrichment analysis of genetic dependencies in melanoma** The most significant biological processes, molecular functions and enriched KEGG 2019 human pathways of 33 significant fitness genes in cutaneous melanoma. The data were filtered for multiple comparisons testing (adjusted p-value <0.01).
